# A Probiotic Preparation Hydrolyzes Gliadin and Protects Intestinal Cells from the Toxicity of Pro-Inflammatory Peptides

**DOI:** 10.3390/nu12020495

**Published:** 2020-02-14

**Authors:** Alessandra Giorgi, Rugiada Cerrone, Daniela Capobianco, Simone Filardo, Patrizia Mancini, Flavia Zanni, Sergio Fanelli, Paola Mastromarino, Luciana Mosca

**Affiliations:** 1 Department of Biochemical Sciences, Sapienza University of Roma, 00185 Rome, Italy; alessandra.giorgi@uniroma1.it (A.G.); rugiadacerrone@gmail.com (R.C.); flaviazanni92@gmail.com (F.Z.); sergiofanelli.bio@gmail.com (S.F.); 2 Department of Public Health and Infectious Diseases, Section of Microbiology, Sapienza University of Roma, 00185 Rome, Italy; daniela.capobianco@uniroma1.it (D.C.); simone.filardo@uniroma1.it (S.F.); paola.mastromarino@uniroma1.it (P.M.); 3 Department of Experimental Medicine, Sapienza University of Roma, 00161 Rome, Italy; patrizia.mancini@uniroma1.it

**Keywords:** gliadin, lactobacilli, bifidobacteria, celiac disease, occludin, zonulin, Caco-2, IL-6

## Abstract

Celiac disease (CD) is an autoimmune enteropathy caused by an intolerance to gluten proteins. It has been hypothesized that probiotic bacteria may exert beneficial effects by modulating inflammatory processes and by sustaining peptide hydrolysis at the intestinal level. This study aims at evaluating the capacity of a probiotic mixture (two different strains of lactobacilli and three of bifidobacteria) to hydrolyze gluten peptides following simulated gastrointestinal digestion of gliadin (PT-gliadin). The capacity of bacterial hydrolysates to counteract the toxic effects of gliadin-derived peptides in Caco-2 cells was also assessed. The protein and peptide mixtures, untreated or proteolyzed with the probiotic preparation, were analyzed before and after each proteolytic step with different techniques (SDS-PAGE, reverse phase HPLC, filtration on different molecular cut-off membranes). These experiments demonstrated that PT-gliadin can be further digested by bacteria into lower molecular weight peptides. PT-gliadin, untreated or digested with the probiotics, was then used to evaluate oxidative stress, IL-6 cytokine production and expression of tight junctions’ proteins—such as occludin and zonulin—in Caco-2 cells. PT-gliadin induced IL-6 production and modulation and redistribution of zonulin and occludin, while digestion with the probiotic strains reversed these effects. Our data indicate that this probiotic mixture may exert a protective role in CD.

## 1. Introduction

Gliadin, a major component of the gluten, comprises a class of prolamines produced by plants of the *Triticum* genus. Gliadin is responsible for the intestinal damage in celiac disease (CD), an autoimmune disease mediated by T-cell activation in the gastrointestinal mucosa [1]. This disease arises in genetically predisposed individuals after exposure to digested gluten. Gliadin peptides, the immunogenic part of gluten, are poorly degraded by the enzymes of the gastrointestinal tract. Several gliadin epitopes show different immunogenic and toxic properties. These epitopes present multiple proline and glutamine residues that give rise to resistance to proteolysis by gastric, pancreatic and intestinal proteases. This results in incomplete digestion of gluten and the generation of immunogenic peptides such as the 33-mer, which has been shown to be relevant for the immune response in celiac disease [2,3].

CD may occur early in life, since the first exposure to gluten, or may develop in later years. In any case, it is a lifelong disease which seriously affects patient’s quality of life. Indeed, the only available therapy for those patients is to avoid ingestion of gluten via the gluten-free diet (GFD). However, gluten may be present as contaminant at very low concentration in many food products due to cross contamination during food processing or cooking, and this may pose serious health risks for celiac patients. Furthermore, many celiac patients even if on a strict GFD still present IBS-like symptoms of the disease (mainly abdominal pain or discomfort in association with a change in bowel habit and often bloating) [4].

Several studies have reported intestinal microbiota perturbations in subjects with CD particularly in patients with persistent symptoms despite adherence to a long-term GFD [5]. In particular, an increase in the abundance of *Bacteroides* [6,7] and a decrease of *Bifidobacterium* spp. [7,8,9] in biopsies and/or stools of active and non-active CD have been consistently found. An increase of Proteobacteria has also been associated with CD but mainly with the active phase of the disease [6,9,10,11]. Whether this imbalance is cause or consequence of the disease is still poorly understood; however, the dysbiosis still persists in patients on a GFD, probably because of the reduction in polysaccharides intake.

Since the intestinal microbiota contributes to maintaining both the intestinal barrier function and the integrity of the intestinal epithelium, this imbalance could have a role in the pathogenesis of CD. Restoring a healthy microbiota in CD patients may provide beneficial effects in these subjects. Supplementation with probiotics is an appealing treatment due to their anti-inflammatory and immunomodulatory effects and to the low risk of adverse reactions. A number of studies were conducted in children affected by CD, to evaluate the efficacy of different probiotic strains, showing positive effect on decreasing the production of pro-inflammatory cytokine TNF-α, in restoring the healthy percentage of main microbial components at the gut level and in modulating the peripheral immune response [12,13,14,15].

A recent clinical trial in adults affected by CD on a GFD with IBS symptoms revealed that a probiotic mixture of lactobacilli and bifidobacteria was effective in reducing the severity of IBS-like symptoms, and was associated with a modification of gut microbiota, characterized by an increase of bifidobacteria and lactobacilli [16]. The probiotic mixture utilized in the clinical trial contained five different bacterial strains, which were isolated from human fecal samples and are endowed with the capacity to colonize the human gut. The aim of the present work was to evaluate the ability of this probiotic mixture to digest gliadin peptides *in vitro* and to modify gliadin-induced pro-inflammatory response and alterations of epithelium structure in human intestinal Caco-2 cells. This epithelial cell line was originally derived from a colon carcinoma and has been extensively utilized as a model of the intestinal barrier [17]. When grown to confluence, Caco-2 cells spontaneously differentiate into a monolayer with many properties typical of absorptive enterocytes (i.e., polarization with brush border layer as found in the small intestine, expression of enzyme activities as those of enterocytes and formation of tight junctions between adjacent cells).

## 2. Materials and Methods

### 2.1. Materials

Gliadin from wheat, pepsin from porcine gastric mucosa, trypsin from porcine pancreas and chymotrypsin from bovine pancreas, ionomycin and Phorbol 12-myristate 13-acetate (PMA) were purchased from Merck-Sigma (Milan, Italy). Gradient grade acetonitrile and water for UPLC were from Carlo Erba reagenti (Milan, Italy). The 33-mer peptide was purchased from GenScript (Hong Kong, PRC), whereas carboxy-dichlorofluorescein diacetate (cDCF) was from Molecular Probes. Cell culture reagents and media were from Gibco BRL (Life Technologies Inc., Grand Island, NY, USA). All other reagents were analytical grade reagents from Merck-Sigma (Milan, Italy).

### 2.2. Bacterial Strains

The probiotic product was provided by NOOS s.r.l. It consists of a mixture of 5 different bacterial strains, namely *Lactobacillus paracasei* 101/37 LMG P-17504 (5 × 10^9^ CFU/sachet), *Lactobacillus plantarum* 14 D CECT 4528 (5 × 10^9^ CFU/sachet), *Bifidobacterium animalis* subsp. *lactis* Bi1 LMG P-17502 (3.4 × 10^9^ CFU/sachet), *Bifidobacterium breve* Bbr8 LMG P-17501 (3.4 × 10^9^ CFU/sachet) and *Bifidobacterium breve* BL10 LMG P-17500 (3.4 × 10^9^ CFU/sachet).

### 2.3. Gliadin Preparation and Proteolytic Digestion

Aliquots (120 mg) of a commercially available gliadin were solubilized with 4 mL of aqueous solution of 0.02 N HCl and submitted to proteolysis by pepsin (3 mg), solubilized in 0.02 N HCl, according to a ratio of 1:40 (gliadin:enzyme, *w*/*w*) for 1 h at 37 °C under gentle stirring. The pH was then adjusted to a value of about 6 by adding a few granules of Tris salts. An amount of 3 mg of trypsin in 50 mM acetic acid was added, according to a ratio of 1:40 (gliadin:enzyme, *w*/*w*). The proteolysis was carried out overnight at 37 °C under gentle stirring to obtain the gliadin digest (PT-gliadin). After digestion, the PT digest was heated at 100 °C for 15 min to inactivate the enzymes and then divided in aliquots for further analyses. Aliquots of this digested gliadin and of untreated gliadin were further incubated overnight at 37 °C in the presence or absence of the probiotic product. Specifically, one sachet of product (containing a total amount of 20 × 10^9^ CFU) was washed centrifuging at 2500*g* for 15 min in 40 mL of sterile PBS to eliminate excipients. The bacterial pellet was resuspended in 10 mL of sterile PBS (final concentration 2 × 10^9^ CFU/mL). One mL of the probiotic suspension was centrifuged at 4600*g* for 10 min and the bacterial pellet resuspended in 2 mL of 30 mg/mL PT-gliadin digest or of untreated gliadin. Samples were then incubated overnight at 37 °C in anaerobic atmosphere under constant shaking to get the PT-gliadin plus bacteria (PT-gliadin+bact) digest and gliadin plus bacteria (gliadin+bact). After incubation bacterial cells were removed by centrifugation (4600*g* for 10 min).

Another aliquot (30 mg) of gliadin, solubilized in 6 mL of 0.02 N HCl, was submitted to chymotrypsin digestion (Chymo-gliadin), after pH adjustment to about 6, according to a ratio of 1:200 (gliadin:enzyme, *w*/*w*), at 37 °C overnight. At the end of treatment an aliquot of this chymotrypsin digested gliadin was incubated overnight with bacteria, as described for PT-gliadin, thus obtaining Chymo-gliadin plus bacteria (Chymo-gliadin+bact).

To verify the capacity of bacterial strains to degrade the 33-mer peptide, 33-mer at a concentration of 1 mg/mL was dissolved in PBS and in de Man, Rogosa and Sharpe (MRS) broth (Oxoid, Milan, Italy), the optimal growth medium for lactobacilli and bifidobacteria. Samples were then incubated overnight with bacteria as described for PT-gliadin.

### 2.4. SDS-PAGE Electrophoresis of Digested Gliadins

Aliquots of 30 µg of samples were separated on an SDS-PAGE Criterion TGX Precast gels (Bio-Rad, Milan, Italy). Coomassie Blue was used to stain the bands and the images were acquired by using the Chemidoc TM (Bio-Rad, Milan, Italy). Densitometry of the lanes from about 55 kDa to the bottom of the lane was performed by using Image J software.

### 2.5. Gliadin Immunological Analyses

Aliquots of 0.5 mL containing 500 µg of PT-gliadin and PT-gliadin+bact were sequentially filtered onto 30 and 3 kDa membrane filters by following manufacturer’s instructions (Amicon, Merck Millipore, Darmstadt, Germany). Before performing ELISA test, samples were extracted from the filtrates with 60% ethanol solution in water according to the manufacturer instructions. Retained and eluted materials were re-diluted with ethanol solution to the initial volume and all the samples were quantified by a competitive ELISA test (RIDASCREEN Gliadin competitive, R-Biopharm AG, Darmstadt, Germany). The competitive ELISA test was used to quantify small peptide fragments of gliadin that cannot be detected by a sandwich ELISA format, in which at least two epitopes are necessary. The limit of detection of the test is 2.3 mg gliadin/Kg corresponding to 2.3 ppm.

### 2.6. HPLC/DAD Analysis of Digested Gliadins

Aliquots of gliadin, PT-gliadin and PT-gliadin+bact were diluted 1:30 in mobile phase A and filtered onto 0.45 µm filters. Aliquots of 200 µL of diluted samples were analyzed by HPLC/DAD on a Waters apparatus consisting of Waters 60F pumps and 600 pumps control unit system equipped with a Symmetry C18 column (300 Å, 5 μm, 4.6 × 250 mm) associated with a guard column of the same material (Waters Corporation, Milford, MA, USA), an inline degasser and a Waters 717 auto-sampler and a spectrophotometric photodiode array detector mod 2996. The mobile phase A consisted of 0.1% trifluroacetic acid (TFA) and mobile phase B was 0.1% TFA in acetonitrile. Gradient elution was performed at a flow rate of 1 mL/min at 25 °C as follows: 90% A for 1 min, to 44% A in 29 min, to 0% A in 1 min. The column was washed with 100% B for 10 min then re-equilibrated to 90% A for 20 min. The instrument control and data acquisition were carried out using the Waters Millennium^32^ software.

### 2.7. UPLC/MS Analysis of 33-mer Peptide Standard or Digested Gliadins

The 33-mer peptide and the peptide mixtures obtained from proteolysis of gliadin were analyzed by a LC-MS platform consisting of an UPLC ACQUITY system (Waters Corporation, Milford, MA, USA), equipped with a C18 column (BEH C18 Column, 130 Å, 1.7 µm, 2.1 × 50 mm), connected on line via an electrospray ion source (Waters Corporation, Milford, MA, USA) to a single Quadrupole (QDa) detector and a spectrophotometric (PDA) detector. Each sample was automatically injected from an autosampler onto the column at a flow rate of 0.5 mL/min at 25 °C. For the elution two solvent solutions were used: aqueous solution with 0.1% formic acid (solvent A) and organic solution (acetonitrile) with 0.1% formic acid (solvent B). The following linear gradient was applied: 90% A for 1 min, to 30% A in 6 min, then 0% A for 2 min.

### 2.8. Culture of Caco-2 Cells

The human colon carcinoma Caco-2 cell line, obtained from ICLC Cell bank (Genoa, Italy), was grown in DMEM high glucose, supplemented with 10% heat inactivated fetal bovine serum, 4 mM glutamine, 100 u/mL penicillin, 100 u/mL streptomycin and 1% non-essential amino acids (Gibco BRL Life Technologies Inc., Grand Island, NY, USA). Cells were maintained at 37 °C in 5% CO_2_ and the culture medium was changed every two days.

For experimental studies, cells between passages 20 to 30 were seeded at a density of 150,000 cells/well in 6-well plates, or on 8-well Lab-Tek chamber slides (Nunc, Merck Millipore, Darmstadt, Germany) at a density of 50,000 cells/well, and grown for 21 days until differentiation. Cell viability was assessed by MTT assay in the presence of gliadin peptides.

### 2.9. Inflammatory Cytokines Detection

Cells grown until differentiation were stimulated with PMA/ionomycin in the presence of PT-gliadin or PT-gliadin+bact. Specifically, cells were pre-treated for 3 h with 1mg/mL PT-gliadin or PT-gliadin+bact, then stimulated with 20 ng/mL PMA and 0.3 ug/mL ionomycin for the next 24 h. After incubation, the cell culture supernatants were collected and assayed for the presence of IL-6 by means of ELISA, following the instructions of the manufacturer (R&D System, Minneapolis, MN, USA).

### 2.10. Oxidative Stress Measurements

Caco-2 cells grown in 6-well plates were loaded with 10 µM cDCF for 30 min in Hank’s Balanced Salt solution, then were treated for 1 h with 1 mg/mL PT-gliadin or PT-gliadin+bact in complete culture medium. A positive control was obtained by using 200 µM H_2_O_2_. After treatment, cells were washed in cold PBS and intracellular fluorescence was assayed with a BD Accuri C6 flow cytometer. A minimum of 50,000 events were recorded for each sample. The experiment was repeated three times in duplicate.

### 2.11. Immunofluorescence of Zonulin and Occludin

Caco-2 cells grown until differentiation on Lab-Tek chamber slides (Nunc, Merck Millipore, Darmstadt, Germany) were treated for 3 h with 1 mg/mL PT-gliadin or PT-gliadin+bact in complete medium, then were washed with PBS and fixed with 2% formaldehyde for 15 min at room temperature. Cells were permeabilized with 0.1% triton, exposed to blocking buffer (5% BSA in PBS) for 30 min, and then incubated for 2 h at room temperature with rabbit polyclonal anti-zonulin (ZO-1) (1:100 dilution) or anti-occludin (1:100) antibody (Thermo Fisher, Waltham, MA, USA) in 1% BSA in PBS. After washing, chambers were exposed for 1 h to the secondary goat anti-rabbit antibody Alexafluor 488 (1:100 dilution) (Abcam, Cambridge, UK). Nuclei were stained for 1 min with 1 µg/mL of 4′,6-diamidino-2-phenylindole (DAPI) in saline solution, and the coverslips were mounted with Mowiol. Fluorescence signal was analyzed by recording stained images using an AxioObserver inverted microscope, equipped with the ApoTome System (Carl Zeiss Inc., Oberkochen, Germany). Microscopy imaging was performed using the Axiovision software (Zeiss).

### 2.12. Statistical Analyses

Experiments were repeated at least three times in duplicate. Data are presented as mean ± S.D. *P*-values have been calculated with the unpaired t-Student test and *P*-values less than 0.05 were considered as significant.

## 3. Results

### 3.1. Proteolytic Activity of the Probiotic Mixture

**Figure 1:**
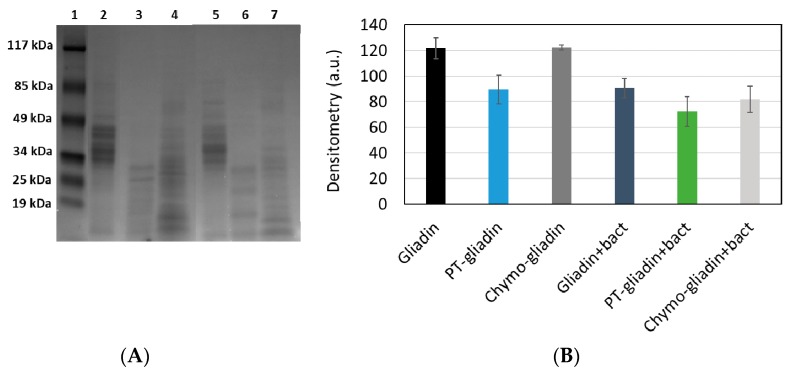
(**A**) Sodium dodecyl sulfate polyacrylamide gel electrophoresis (SDS-PAGE) analysis of gliadins. (1) Molecular weight (MW) markers; (2) Gliadin standard; (3) PT-gliadin; (4) Chymo-gliadin; (5) Gliadin+bact; (6) PT-gliadin+bact; (7) Chymo-gliadin+bact. (**B**) Densitometry of the lanes from about 55 kDa to the bottom of the lane performed by using ImageJ software.

In order to assess whether the probiotic mixture was able to further proteolyze gliadin after a first enzymatic digestion by a combination of pepsin and trypsin (PT) or by chymotrypsin alone, various analytical methods were set up. Figure 1A shows the separation by SDS-PAGE of gliadin (lane 2), PT-gliadin (lane 3) and chymo-gliadin (lane 4) as such or digested with the probiotic mixture (lanes 5, 6 and 7, respectively). Gliadin (lane 2) shows five predominant bands with molecular weight ranging between 35 and 50 kDa, whereas both PT-gliadin (lane 3) and chymo-gliadin (lane 4) clearly show bands with a molecular weight lower than 35 kDa. The protein bands after bacterial treatment show lower molecular weights. The densitometric analysis of the lanes is reported in Figure 1B and clearly highlights that after PT or chymotryptic digestion and further digestion with bacteria, the intensity of the bands is reduced, indicating an extensive proteolysis of the protein. Moreover, the untreated gliadin (lane 2) seems to be partially digested by bacteria (lane 5).

**Figure 2:**
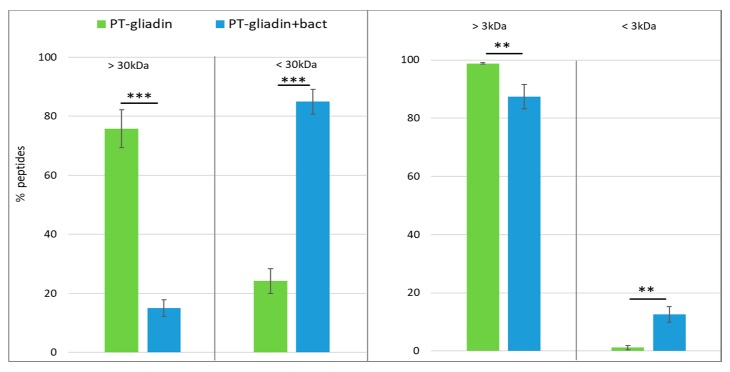
Molecular fractionation of digested gliadin. PT-gliadin and PT-gliadin+bact were sequentially filtered onto membrane filters of 30 and 3 kDa and the amount of peptides assessed by ELISA. ** *p* < 0.01; *** *p* < 0.001.

The same samples were subjected to sequential molecular fractionation by the use of two different membrane filters with a cut-off of 30 and 3 kDa. As shown by a sensitive R5 antibody based competitive ELISA, PT-gliadin is largely retained by 30 kDa cut-off filters, while over 80% of PT-gliadin+bact is found in the filtrate. Moreover, further filtration with 3 kDa cut-off membrane demonstrated that more than 12% peptides from PT-gliadin+bact have a molecular weight lower than 3 kDa (Figure 2).

**Figure 3:**
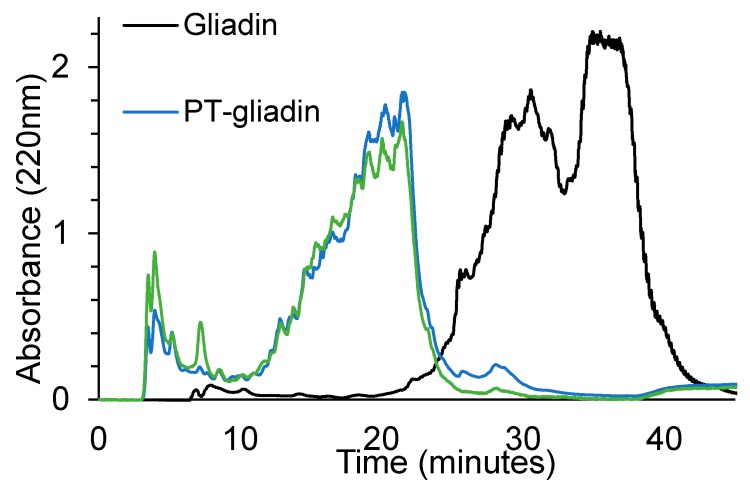
Reverse phase HPLC of Gliadin (black line), PT-Gliadin (blue line) and PT-gliadin+bact (green line).

A similar pattern is exhibited when gliadin, PT-gliadin and PT-gliadin+bact were analyzed by reverse phase HPLC (Figure 3). Gliadin presents a large peak with retention time between 25 and 40 min, which clearly shows contribution from the different protein subtypes. In PT-gliadin the chromatographic profile is totally different and there is one major contribution of a chromatographic signal with retention time between 10 and 25 min, and a small contribution of peptides which are less hydrophobic (presumably with a lower molecular weight) and have retention times between 3 and 10 min. The further digestion with bacteria clearly enhances the amount of less hydrophobic peptides. Particularly, a peak with a retention time of 7 min can be evidenced, which is present only in trace amount in the PT digest.

**Figure 4:**
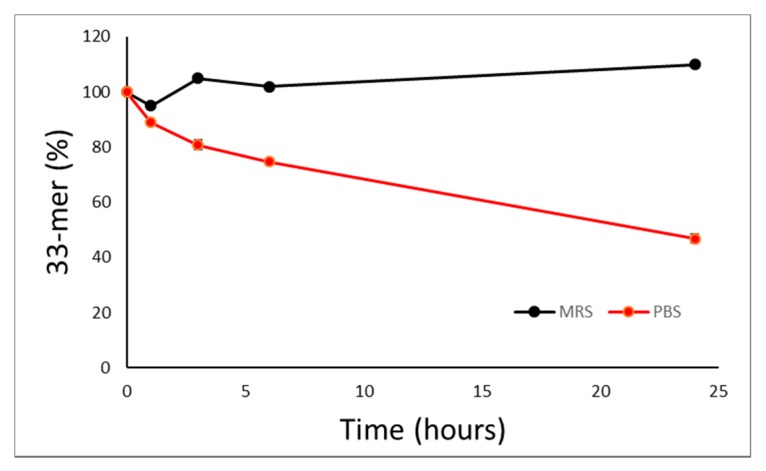
Kinetics of 33-mer digestion in de Man, Rogosa and Sharpe broth (MRS) or in phosphate buffered saline solution (PBS). A total of 1 mg/mL 33-mer peptide was incubated in the presence of 10^9^ CFU/mL at 37 °C up to 24 h. The amount of peptide was quantified via ultraperformance liquid chromatography with diode array and mass detectors (UPLC/DAD/MS).

The same peptide mixtures were also analyzed by UPLC//MS/DAD platform, applying analytical conditions optimized for 33-mer standard peptide. Analyses of PT-gliadin allow to identify, among others, a peptide with an *m*/*z* signal at 802.3, also described by Laparra et al. [18], but no peptides corresponding to the 33-mer peptide could be evidenced. Conversely, the chromatographic profile of chymo-gliadin allows to identify a *m*/*z* signal at 978.2, at the same retention time of [MH^+4^] of 33-mer peptide, i.e. 4.40 min. The 33-mer peptide was analyzed by this MS platform after probiotic treatment. Figure 4 shows the kinetics of 33-mer digestion after incubation in the presence of the probiotic mixture. It can be observed that when the 33-mer peptide is incubated with bacteria in MRS culture medium, which confers adequate nutrients to the bacteria, the 33-mer peptide remains unaltered. However, when the peptide is incubated in PBS, its amount is reduced by about 50% in the presence of bacteria after 24 h incubation, indicating that they can degrade this peptide, though not completely.

**Figure 5:**
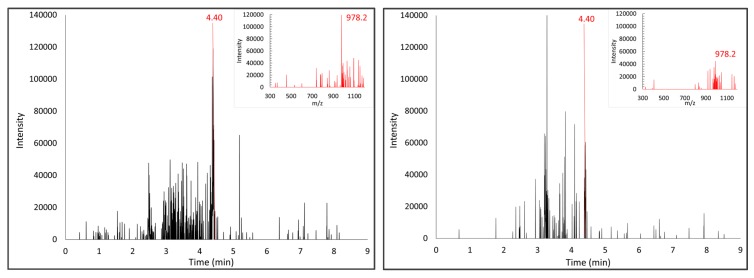
Left panel: Chromatographic profile and the mass spectrum of [MH^+4^ = 978,2] of 33-mer peptide in chymo-gliadin. Right panel: Chromatographic profile and the mass spectrum corresponding to the same retention time of expected [MH^+4^ = 978,2] of chymo-gliadin after probiotic digestion.

Figure 5 reports the results of the analyses of the chymo-gliadin, revealing the presence of the peak corresponding to 33-mer peptide at 4.40 min, and the attenuation of *m/z* signal corresponding to [MH^+4^] of 33-mer peptide after probiotic digestion.

### 3.2. Effect of PT-Gliadin and PT-Gliadin+Bact on the Caco-2 Tight Junctions

Since the main function of the intestinal epithelium is that of a selective barrier regulating the passage of various substances into the subepithelial compartment, here we examined the integrity of the tight junctions by analyzing the localization of zonulin (ZO-1) and occludin, the best studied components of the junctions. To this aim, we utilized the intestinal epithelial cell line Caco-2, that is widely used as a model for the in vitro study of human intestinal epithelium functions. We performed immunofluorescence analyses to establish whether the ZO-1 release and occludin rearrangement following PT-gliadin treatment would be prevented by further digestion with bacteria.

**Figure 6:**
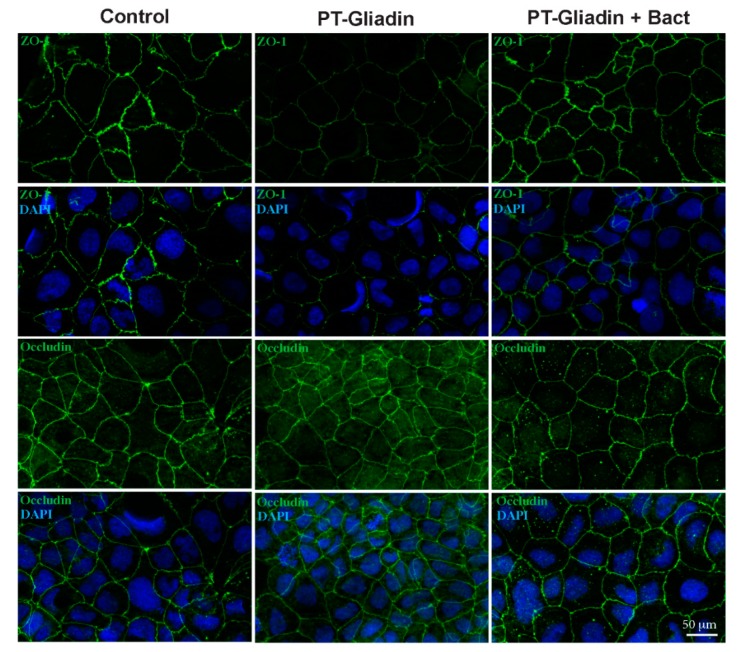
Caco-2 cells stained for zonulin (ZO-1) or occludin. Cells were treated with 1 mg/mL of PT-gliadin or PT-gliadin+bact and, after 3 h of treatment, cells were fixed and stained with antibodies against ZO-1 or occluding, and with 4′,6-diamidino-2-phenylindole (DAPI) to counterstain nuclei. Bar, 50 μm.

Our results show that untreated Caco-2 monolayers had the typical ZO-1 and occludin localization at the cell periphery [19], whereas the treatment with PT-gliadin for 3 h induces a marked reduction of ZO-1 fluorescence and a redistribution at the cytosolic level for occludin. When cells were treated with PT-gliadin+bact, neither ZO-1 release nor occludin relocation could be observed, although an increase in punctate cytoplasmic staining of occludin was evident (Figure 6).

### 3.3. Modulation of IL-6 Production and Oxidative Stress by Caco-2 Cells Exposed to PT-Gliadin Digested or Not with Bacteria

**Figure 7:**
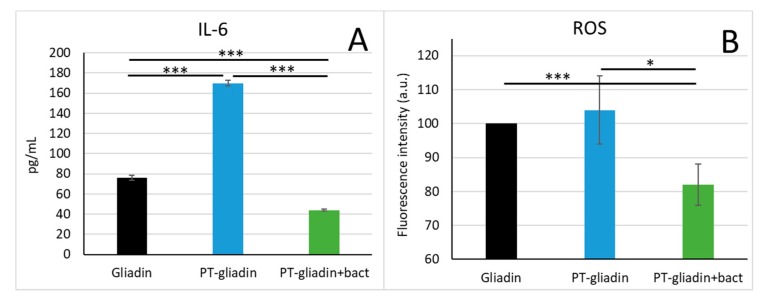
(**A**) Interleukin 6 (IL-6) production in Caco-2 cells stimulated with PMA/ionomycin in the presence of gliadin peptides. Cells were exposed to 1mg/mL PT-gliadin or PT-gliadin+bact, then supernatants of cell culture were analyzed by ELISA. (**B**) Intracellular reactive oxygen species (ROS) as determined by flow cytometry. Cells were exposed to 1mg/mL PT-gliadin or PT-gliadin+bact, stained with cDCF and then analyzed by flow cytometry. * *p* < 0.05; *** *p* <0.001.

Caco-2 cells were also assessed for the production of IL-6 when stimulated with non-cytotoxic concentration of gliadin peptides. The PT-gliadin was able to cause a significant increase in IL-6 production compared to control. Conversely, when the cells were stimulated in the presence of PT-gliadin+bact no increase in IL-6 could be observed, instead a significant reduction in IL-6 production compared to control cells was observed (Figure 7A).

In order to verify whether digestion with probiotic bacteria could modulate the pro-oxidant effects of PT-gliadin, Caco-2 cells were treated with PT-gliadin or PT-gliadin+bact and then assessed for intracellular hydroperoxides (Figure 7B). In our experimental conditions after PT-gliadin treatment no significant evidence of oxidative stress could be observed; however, treatment of cells with PT-gliadin+bact caused a significant reduction of intracellular reactive oxygen species, indicating that the probiotic digest reduces the basal level of oxidative stress in the cells.

## 4. Discussion

The intestinal microbiota is a complex ecosystem in which thousands of bacterial species are present and play a key role in the host’s health. Intestinal microbiota is involved, among other functions, in the maturation, maintenance and behavior of the mucosal immune system and contribute to the digestive process of food in a symbiotic manner [20]. Bacterial species may express enzymes different than those produced by humans and may favor the digestion of complex carbohydrates, fiber in particular, but also of proteins.

The potential use of probiotics in CD management is supported by the intestinal dysbiosis generally associated with CD [5,6,7,8,9,10,11] and the role attributed to these beneficial bacteria in degradation of toxic compounds, in maintaining gut barrier function and in regulating the response of the innate and adaptive immune system. The results of our study indicate that selected strains of lactobacilli and bifidobacteria are able to hydrolyze the gliadin fragments generated by digestive proteases into smaller peptides. We demonstrated that the treatment of PT-gliadin fragment mixture with probiotic bacteria is able to significantly decrease the molecular dimension of these fragments, as highlighted by different kind of experiments, such as the SDS-PAGE, HPLC measurements and molecular fractionation experiments. In particular, as estimated by a specific ELISA assay, the amount of peptides with molecular weight lower than 3 kDa is markedly higher in PT-gliadin+bact in comparison to PT-gliadin. Moreover, the probiotic strains were able to reduce the amount of the 33-mer immunotoxic peptide when this compound was the sole amino acidic source. This confirms that the selected bacteria have a specific transport system to intake immunogenic oligopeptides, as already suggested for other bacterial strains [21].

Some previous in vitro studies have demonstrated that the proteolytic activity of bacteria may modify gliadin fragments [18,22,23]. The presence of some Bifidobacterium strains *(B. bifidum* IATA-ES2, *B. longum* IATA-ES1, and *B. animalis* IATA-A2) during the intestinal digestion of gliadin led to the generation of different fragment sequences in vitro, with lower molecular mass than those generated in non-treated samples [18], as observed in our study. The CD immunogenic 33-mer peptide was degraded and lost its antigenicity in both in vitro and in vivo assays when it was exposed to a bacterial prolyl endopeptidase derived from *Flavobacterium meningosepticum* [24]. It has been demonstrated that the combined activity of different peptidases promoted the hydrolysis of 33-mer. Overall, five peptidases are required to completely degrade the 33-mer and other synthetic immunogenic peptides [25]. A recent study identified 10 strains of lactobacilli that, when pooled, provided the peptidase portfolio required to completely degrade the immunogenic gluten peptides, including the 33-mer, involved in CD [26]. A consortium of different strains was necessary to obtain this result because no single bacterial strain possesses all the peptidases required to completely hydrolyze gluten immunogenic epitopes [25,27,28].

Noteworthy, the results observed in our study were obtained with a multi-strain probiotic preparation containing two strains of lactobacilli (*L. paracasei*, *L. plantarum*) and three strains of bifidobacteria (two different *B. breve* and *B. animalis*) that probably exert complementary effects on the hydrolysis of gliadin peptides. The biological effect of proteolysis of gliadin by probiotic bacteria was analyzed in relation to intestinal permeability, inflammation or oxidative stress on the intestinal cells. Our results clearly show that the PT fragments from gliadin are able to induce tight junction destabilization and an inflammatory state on intestinal Caco-2 cells. On the other hand, the low MW fragments generated after bacterial treatment seem to prevent the disruption of tight junction proteins, antioxidant and anti-inflammatory activities, overcoming the negative effect induced by untreated PT-gliadin. The reversion of IL-6 increased production exerted by gliadin that was induced by probiotic digestion of PT fragments appears of great interest. Indeed, in CD patients, gliadins induce the production of pro-inflammatory cytokines, among others IL-6, which stimulates growth of T cells and differentiation of cytotoxic T cells and has been suggested as a marker of both CD activity and response to treatment [29,30].

Other probiotic strains have shown the capacity to reverse the negative effect exerted by gliadin on the barrier function of intestinal cells and inflammation. *Bifidobacterium animalis* ssp. *lactis* was able to counteract the gliadin-induced change in tight junctional protein ZO-1 expression [31], and *Lactobacillus rhamnosus* GG contributed in vitro to the maintenance of normal permeability in Caco-2 cell cultures exposed to gliadin [32]. Some Bifidobacterium strains (*B. bifidum* IATA-ES2, *B. longum* IATA-ES1, and *B. animalis* IATA-A2) reduced the production of TNF-α induced in cultured intestinal cells by gliadin peptides generated during gastrointestinal digestion [18].

It is well known that oxidative stress and inflammation are closely related phenomena and that gliadin-derived peptides are able to induce oxidative stress in Caco-2 cell cultures [33]. Our results indicate that the digestion of gliadin peptides with probiotic bacteria can induce an antioxidant effect which can result in a modulation of the intracellular redox balance, thus protecting the cells from the toxic effects of PT-gliadin.

Promising results could come also by chymotryptic proteolysis experiment, because our preliminary evidences indicate that the probiotic product is able to modulate the amount of the 33-mer peptide generated by chymotrypsin digestion. This could be reasonable, considering the presence of different kind of proteases in the intestinal tract, each according to its specificity, to proteolyze the proteins in smaller fragments. Our results are in line with previous publications which demonstrate that probiotic lactobacilli and bifidobacteria may digest gluten peptides, thus reducing their immunogenic potential [18,26,34]. Most notably, these properties may well justify the beneficial effects observed after administration of the same probiotic product studied in our paper to CD patients affected by IBS-like symptoms even if on a GFD [16], and reinforce the concept of the therapeutic potential of probiotics in CD.

## 5. Conclusions

In conclusion, our results suggest that the probiotic strains tested are able to reduce the toxicity of gliadin that remains after peptic-tryptic digestion by degrading immunodominant gliadin peptides, thus inhibiting their noxious effects on intestinal epithelial cells. The results obtained from our in vitro studies could have a higher value if verified in animals. In fact, recent work has identified an animal model of gliadin-induced enteropathy which has made it possible to confirm the beneficial effects of probiotic bacteria strains observed in vitro. Indeed, the administration of *B. longum* CECT 7347 (previously named IATA-ES1) has been shown to reduce the production of inflammatory cytokines and the CD4+ T-cell mediated immune response in this animal model [35], while LGG was able to protect the intestinal mucosa of rats from PT-gliadin-induced damage, by preventing the reduction of the expression of the intercellular junction proteins [36].
